# Quality of intrapartum care at health centers in Jabi Tehinan district, North West Ethiopia: clients’ perspective

**DOI:** 10.1186/s12913-020-05321-3

**Published:** 2020-05-19

**Authors:** Kerebih Asrese

**Affiliations:** Faculty of Social Sciences, Bahir Dar University, Bahir Dar, Ethiopia

**Keywords:** Facility delivery, Intrapartum care, Quality, Experience, Ethiopia

## Abstract

**Background:**

Despite progress recently, Ethiopia remains one of the largest contributors to the global burden of maternal deaths. As facility-based childbirth and skilled-birth attendant at birth reduces maternal morbidity and mortality, the country has been implementing expansions in infrastructure during the past decades. Whether this phenomenal expansion in infrastructure and improvement in coverage of healthcare services matched with quality of maternal health service is not well investigated. This study assessed the quality of intrapartum care experienced by mothers at health centers in Jabi Tehinan district, North West Ethiopia.

**Method:**

An institution-linked community-based cross-sectional study was carried out from August to September 2018 to assess the quality of intrapartum care experienced by mothers during facility deliveries. Quantitative data were collected from 378 women who had uncomplicated births at health centers within 6 months preceding the survey and interviews were held with 25 women. The quantitative data were entered into SPSS for Windows versions 23 for analyses. Qualitative data were transcribed verbatim and data were used to substantiate the quantitative data.

**Result:**

The results indicated that 13% of mothers experienced good quality of intrapartum care services. About 49, 45, 31, and 25% of mothers respectively experienced good quality of human and physical resources; respect, dignity, and equity; emotional support; and cognition. Only 2% of mothers experienced good quality of services on the four dimensions and 15% did not experience good quality of services on neither of the dimensions. Mothers from rural areas and mothers who did not use ANC services for recent born children were found more likely to experience good quality of intrapartum care. Informants discussed poor quality of labor environment, lack of privacy, and poor client-provider communications at health facilities.

**Conclusion:**

The results indicated that the quality of intrapartum care experienced by mothers were minimal. The findings highlighted that efforts exerted to increase infrastructure and improve maternal health service coverage did not ensure quality maternal health services. Therefore, to increase the uptake of facility births and improved maternal health outcomes, stakeholders should make the health system humane, respectful, equitable, and responsive to mothers’ concerns.

## Background

Pregnancy and childbirth-related complications continue to be risks for morbidity and mortality among women of reproductive age in developing countries [1]. Worldwide, an estimated 295,000 women died in 2017 from pregnancy and childbirth-related causes, with Sub-Saharan Africa accounting for 66% of the global burden. With estimated 16,000 maternal deaths in the same year, Ethiopia is one of the Sub-Saharan countries with highest number of maternal mortality [2].

Because the major causes of maternal death are preventable [3], efforts to reduce maternal morbidity and mortality emphasize facility-based childbirth and skilled attendance at birth and timely referral for emergency obstetric care if complications occur [4]. This priority is echoed in the Sustainable Development Goals to end preventable maternal mortality and reduce global maternal mortality ratio to less than 70 per 100,000 live births [2]. In line with this global initiative, the Government of Ethiopia committed to reduce maternal mortality ratio to 199 per 100,000 live births by 2020 [5].

To meet this national goal for maternal health, the Government of Ethiopia has been implementing phenomenal expansion in infrastructure such as training and deployment of skilled professionals, construction and expansion of new health centers and hospitals, renovation and maintenance of existing health institutions, and expansion of emergency obstetric care services during the Millennium Development Goals period. In its current health sector transformation plan, the country put unprecedented emphasis on improving quality of care [5]. Whether this phenomenal expansion in infrastructure and improvement in coverage of healthcare services during the Millennium Development Goals period in the country matched with expected quality of service needs investigation. Assuring the quality of care provision during labor, childbirth and immediate postpartum period is of utmost importance for mothers and their newborns health outcomes [6].

Using a facility audit checklist and clinical records, a few authors [7–9] assessed the quality of delivery service provisions in health facilities in different parts of the country and reported that the qualities are suboptimal. As the focuses of these studies were on the status of institutions providing the services and the expertise of the service providers, the quality of services from the clients’ perspectives was not investigated. Users evaluate and define the quality of care based on their current or previous experiences with the health system and those of people they know [10, 11]. Women’s own views on the quality of delivery services they experienced are important for acceptance and sustained use of health services [12, 13].

Defining the quality of maternal health care is debatable, partly due to the inherent complexities of measuring the concept [12]. Early attempts to define and measure the quality of maternal health services were based on the quality of health framework developed by Donabedian [14], which constitutes three aspects of health service delivery: structure, process, and outcome. Structure refers to inputs in the setting where health services are provided (materials, infrastructure, and human resources). Process refers to the technical issues during health service delivery. Outcomes refer to health status and client satisfaction. In defining and measuring quality in the context of maternal health services provided at health facility, Hulton and colleague’s [15] framework separates quality into two constituent parts: quality of the provision of care within the institution and quality of care as experienced by users. The framework identifies 10 elements in assessing the quality of maternal health services, six of which relate directly to the provision of care by the institutions and the other four elements are related to women’s experience of care in the institution.

Previous attempts for evaluating the quality of maternal health services in the country [7–9] used the Donabedian model [14]. In this study, we used Hulton and colleague’s [15] framework to assess the quality of the maternity care from the points of views of mothers who had received intrapartum care services at health centers in Jabi –Tehinan woreda, North West Ethiopia. The objectives of the study were to examine clients’ perspectives on the quality of intrapartum care now on offer and to solicit their views on topical issues of maternity care. The findings will provide relevant information for service providers, decision makers, and other stakeholder on how well the institutions are functioning in providing client-centered delivery services and which aspects of care need to be improved.

## Methods

### Study setting and design

An institution-linked community-based cross-sectional study was carried out in Jabi Tehinan district, Amhara Region, North West Ethiopia from August to September 2018 to assess the quality of facility delivery services experienced by women. Administratively, the district is divided into 3 urban and 38 rural kebeles. About 218, 753 people are living in the district. There are 12 health centers and 41 health posts providing health services for the population including maternal health services. The maternity units in the health centers are staffed and led by midwives. At the time of the survey, all health centers have one or more skilled birth attendants (midwifery) providing delivery services. The midwives have considerable autonomy in their clinical practice- providing care in uncomplicated cases and making referral decisions when and if they consider it necessary. Maternity service is given free of charge in all health centers and the service is supposed to be open 24 h. In 2017, 6639 women gave birth at the 12 health centers [16]. Four health centers were selected randomly and the study was conducted within the catchment area of these health centers.

A sequential explanatory mixed methods design was employed [17]. The qualitative data complemented the survey data by providing an elaborative aid to understanding the statistical patterns that emerged. Data collection with both methods was linked by addressing the same substantive issue of the quality of delivery services experienced by women during child birth (Additional file 1). “The more that the items overlap or complement each other; the more that the mixed methods can be part of a single study” [18]. Integration occurred at the data interpretation stage and in the discussion [17, 18]. This design is useful for explaining and elaborating quantitative findings [19, 20].

### Participants of the study

Women immediately postpartum can be overwhelmed by feelings of exhaustion and relief and may not properly reflect on their experience until much later [21] or may be influenced by courtesy bias, hence may not feel comfortable reporting a negative experience while still at the facility [22]. Thus, lists of women who delivered in the selected health centers within 6 months preceding the survey were obtained from the delivery registration books. This time frame was considered as optimal to reduce recall bias [23]. Then women who had uncomplicated deliveries were selected for inclusion in the study. As outcome of pregnancy may influence perception of quality of care received, women who were severely ill or did not have living infants were excluded from the study. Participants in the qualitative phase of the study were selected purposely considering their parity, age, and duration (in month) since their last birth.

### Sample size and sampling

A single population proportion formula was used to estimate the sample size with assumptions of 5% precision, 95% confidence, and a 10% non-response rate. A study in three referral hospitals in Amhara region [24] reported that about 62% of women were satisfied with the services during child birth. Jabi Tehinan is one of the districts in the region. Therefore, this proportion was used to obtain a proxy estimate of the sample size required to assess the level of quality of delivery services experienced by women during facility-based childbirth. The calculated sample size was 398. Eight women who reported illness following delivery and three women who experienced infant death were excluded from the study. Data obtained from nine women were not complete, hence excluded. Therefore, data obtained from 378 women (about 95% response rate) are presented.

The sample size was proportionally allocated to the selected health centers based on the number of uncomplicated deliveries occurred in the facilities within 6 months preceding the survey. Then systematic random sampling technique was used to select participant mothers from each health center catchment area. The residences of selected mothers were traced using kebele (small administrative unit) addresses filed on institutional delivery service registration books. Health extension workers and Women Development Army Leaders in those kebeles served as data collectors’ guide to show the households of mothers included in the study.

Twenty five women participated in the qualitative phase of the study. These women were selected to share their birth experiences- the care received from the start of their labor to the immediate post natal period (before discharge). Saturation of information was used to decide on adequacy of the sample size for the qualitative inquiry.

### Data collection

We developed structured questionnaire and interview guides based on framework developed to investigate the quality of institutional maternal delivery services as experienced by mothers [15], considering previous literature reporting measurement of delivery service quality perceptions in the country [8, 24], and national standard developed to transform the quality of health care in Ethiopia [25]. The questions comprised of 20 items in four dimensions: 8 items on physical and human resources; 5 items on cognition; 3 items on respect, dignity, and equity; and 4 items on emotional support. The items in each dimension have binary yes/no responses (coded 1 = yes- indicating experienced care and 0 = no- indicating the absence of care). Other variables (socio-economic, demographic, and obstetric characteristics) are included as additional information (Additional file 1).

We initially developed the tools in English and then translated into Amharic (local language) to ease understanding. Prior to the main study, a pretest was conducted on 20 mothers (not included in the main survey) who had uncomplicated deliveries in a comparable health facility to improve clarity, understandability, and simplicity of the messages. Six female data collectors with previous data collection experience collected the quantitative data.

We used semi-structured interview guide to collect qualitative data. The interview items in the guide addressed similar concerns- the quality of delivery services (human and physical resources; cognition; respect, dignity, and equity; and emotional support) experienced by women during labor, delivery and immediate post natal period at health facilities. Additional probing items were also raised during the discussion (Additional file 1). The interviews were audio recorded. Two female research assistants (midwives) not affiliated to the selected health facilities collected the qualitative data.

The research team participated in a two- day intensive training on the study protocol, contents of the questionnaire, and informed consent procedures. Completed questionnaires were checked for completeness on a daily basis. The author monitored the overall data collection process.

### Data analysis

All returned questionnaires were checked for completeness and consistency of responses manually. After cleaning, raw data were entered into SPSS for Windows versions 23 for analyses. Frequencies and percentages were computed to describe major findings of the study. The sum of valid responses for each item in each dimension was computed to obtain the summary measure of each dimension. Mothers were considered as having experienced good quality of care on the four dimensions if they reported yes on 75% or more of the quality indicators assessed in each dimension of care. The sum of valid responses for all items in the four dimensions of care was aggregated and computed to obtain the summary measure of the overall quality of intrapartum care services experienced by mothers. And mothers who reported yes on 75% or more of the items were considered as having experienced good quality of intrapartum care services [7], otherwise classified as having experienced poor quality of care. Chi-square test of independence was also computed to assess the existence of significant association on the level of overall quality of delivery services experienced among women by their background characteristics.

Qualitative data were transcribed verbatim. The transcription of data was undertaken on the same date of each interview. The transcript of each interview was read and re-read thoroughly to understand the content and concepts shared during the interview and to look for differences and similarities in the data [26]. Data were then coded and categorized into themes and integrated into the quantitative findings around the four dimensions of Framework for Evaluation of Quality of Care in Maternity Services [15]. This approach is appropriate when the qualitative data are collected to provide further understanding of the quantitative findings [27].

### Ethical consideration

The study received ethical approval from Ethical Review Committee of Bahir Dar University. Two weeks before data collection, we communicated the selected health institutions and ‘kebele’ administrations with formal letters obtained from Bahir Dar University. Verbal consent of individual participants was obtained after being fully informed of the study purpose and procedures. Women were informed that participation was voluntary and that their participation or lack thereof would in no way impact the care they or their family members would receive now or in the future. Participants were also informed that they might end their participation at any time. We ensured confidentiality by removing all personal identities from the questionnaire.

## Result

### Background characteristics of respondents

The respondents’ age ranged from 18 to 38 years old with mean age 26.7 years. As indicated in Table 1, majority of the mothers (56.6%) are 26 years and above. Nearly two third of the mothers (63%) are from rural areas, 96% are married, and about 60% cannot read and write.

**Table 1:** Percentage distribution of respondents by background characteristics, *n* = 378

Background characteristics	n (%)
**Age**^**a**^	26.76(4.50)
**18–25 years**	164(43.4)
**> = 26 years**	214(56.6)
**Residence**	
Rural	238(63)
Urban	140(37)
**Marital status**	
Divorced	15(4)
married	363(96)
**Educational status**	
No education	225(59.5)
Primary education (grades 1–8)	87(23)
High school and above (> = grade 9)	66(17.5)
**Parity**	
One	112(29.6)
Two to four	206(54.5)
Five and above	60(15.9)
**ANC use for the recent birth**	
No	69(18.3)
Yes	309(81.7)
**Intension of place of delivery**	
Home	42(11.1)
Health facility	336(88.9)

^a^mean (sd)

Three in 10 mothers are primipara (gave birth for their first baby). About 54% of mothers are multipara (gave for two to four births) and 16% of mothers are grandmultipara (gave for five and above births). More than eight in 10 mothers (82%) had antenatal care (ANC) follow up for their recent born children. Of all respondents, 89% planned to deliver at health facility (Table 1).

### Quality of intrapartum care

The quality of care women experienced in health facilities during labor and delivery were assessed considering their contact with and experience of human and physical resources; their cognition (the level to which she understands what is happening to her and why); the respect, dignity, and equity of care they receive in their stay in the facilities; and the emotional support they receive during labor and delivery. Women were asked retrospectively their experience, i. e., the presence/absence of these cares during labor and delivery at health facilities for their most recent borne children in line with these four dimensions.

#### Contact with human and physical resources

A woman’s experience of care of this dimension was assessed by considering her experience of and contact with attendants and her perception of the quality of the laboring environment (the bed, delivery room, toilets, water, and electricity). More than 90% of the respondents reported that the attendants were available in the facility when they wanted them (Table 2). Similar experience was also shared by qualitative informants. Nineteen out of 25 informants described that health professionals were available in the facilities while they were in the institutions. For six mothers who went for delivery during late afternoon or night, the attendants were not around. The security guards called attendants for them.

**Table 2:** dimensions of care and types of care experienced by women during uncomplicated births at health facility, *n* = 378

Experienced quality Dimensions	Sub-components of the dimension measured	Yes n(%)
**Human and physical resources**	Attendant is available in the facility	341(90.2)
	Clean and good-looking labor ward	309(81.7)
	Available electric light in the facility	306(81)
	Labor room is not crowded	264(69.8)
	Clean delivery beds/coach	220(58.2)
	Clean delivery room	202(53.4)
	Water is available in the facility	189(50)
	Clean toilet in the facility	164(43.4)
**Cognition**	Initial assessment and health history taken at arrival	359(95)
	The diagnoses were explained	154(43)^a^
	Caring procedures were explained to mother	98(25.9)
	Attendants answer mothers’ questions	103(27.2)
	Advised health promotion (breast feeding, diet, immunization, etc.)	253(66.9)
**Respect, dignity, equity**	Communicate politely while giving services	267(70.6)
	Curtains/barriers were used for privacy	192(50.8)
	Took consent/permission prior to any procedure	88(23.3)
**Emotional support**	Showed compassion for mother’s concerns	274(72.5)
	Supportive staff	177(46.8)
	Companions of mothers’ choice are allowed	8(2.1)
	Gave periodic updates on status and progress of labor	134(35.4)

^a^women who reported that examination and health history was taken at arrival

Women’s experiences of the quality of the laboring environment were mixed. While more than 81% of respondents reported clean and good-looking labor ward, only 58 and 53% of respondents reported clean delivery beds and clean delivery rooms, respectively. Eighty one percent, 50, and 43% of respondents respectively experienced available electric light, available water, and clean toilets in the health facilities. And about 70% of the respondents reported that the delivery rooms are not crowded (Table 2).

Informants viewed the quality of laboring environment in a similar way. While some informants commented the cleanliness of the environments, others were discontented by the adjacent classes for different purposes and the size and adequacy of rooms serving different purposes. Seventeen out of 25 informants shared that there was bad odor in the laboring wards. Informants maintained that though delivery beds looked cleaned, they experienced discomfort when they lay on it due to associated odor in the rooms. The delivery rooms and the prenatal wards are adjacent classes, more often divided by walls where sound disturbance is threatening. A mother, who was in a prenatal ward and listening a crying woman in the delivery room stated her experience that, “when she was shouting and begging support calling her mom, I started to cry without any apparent contraction expecting that I will be the next to experience such pain.” She stated that such experience weakened her emotionally before true labor.

Other informants also described that the prenatal wards are serving different purposes and very crowded. Such contexts compromised their comfort while they were in the institutions. One of the informants explained that:

*… the room is serving dual purposes; it is prenatal ward for laboring women till they will go to the delivery room, it is a post natal room (waiting room for recently delivered women before discharge), and a discussion room between the attendants and mothers. The room is also often visited by women’s companions. It is very crowded and suffocated. You could not feel safe because of disturbances* (mother, 30’s – 40’s).

The narrations revealed that mothers experienced unsatisfying prenatal wards because of its diverse purposes; serving as waiting room for a laboring woman with may be in labor pain, waiting room for post natal women may be overwhelmed by feelings of exhaustion and relief, and discussion room between the attendants and mothers. Services within such environments may not satisfy clients’ expectations.

#### Cognition

Cognition is the extent a woman experienced that she understood what was happening to her and she receives information in understandable manner. In this study, provider-client information exchange seems limited. More than 9 in 10 mothers (about 95%) described that their health status and obstetric condition was assessed at arrival, of which only 43% were informed about the diagnoses. Attendants explained the caring procedures for 26% of the mothers and about 67% of mothers were advised health promotion practices (personal hygiene, diet, immunization, breast feeding). Only 27% of mothers reported that the attendants answered their questions (Table 2).

The qualitative data are in consonant with the overall quality of cognition experienced by mothers. Though all informants stated that their health status and obstetric conditions were examined as soon as they reached the health facilities, none of them were informed a diagnosis that indicate either their health status or obstetric conditions, except information given to be admitted for follow-up or appointment for other visits. The data pointed out that debriefing the diagnoses for laboring mothers seems unusual in the study setting.

Regarding the information explained for mothers about the different caring procedures undertaken for them during the birthing process, mothers’ experiences were very limited. Even, they did not remember what was told to them by the attendants at different phases of labor and during delivery. On probing, all informants reported that they were informed to lie on the back on bed for examination (abdominal palpation, blood pressure check-up, and fetoscope auscultations). They described that they used to be in a different positions/behaviors based on the attendants’ requests. Communications between the attendants and the clients was also very limited. An informant who went to a facility during night explained her experience as follows:

*The attendant was not around when we reached there. Of course, the security guard called her … she came after some minutes (*difficult to guess*). It appears that she came from deep sleep, she did not talk well. She took me to a room where she laid me on the back and touch my belly. She put on gloves and put her fingers into my vagina. She said … it is too early to come to facility, no other information. She called the security guard and said to him something; and went somewhere* (mother, 20’s – 30’s).

Some informants also explained such scenario in a different way. They argued that the attendants are indifferent of women’s concerns. They did not want to talk with mothers. They often did examination and left women without information. They often show bossy behavior. Such context made women in labor to be more anxious to know their status. Regarding this context, a mother explained that, “she spoke with me only a few words. She covered her hand with plastic (glove) and put it into my vagina. Then she went out. I hesitated to ask questions about … , however, I afraid of her response” (mother, 30’s – 40’s).

#### Respect, dignity and equity

This dimension of care refers to a woman’s perception of the manner she was treated by the provider during the birthing process. The survey findings revealed that 71% of mothers had polite communication from the attendants during labor and delivery and 51% reported that curtains or other barriers were used to protect their privacy during examination or childbirth. Consent or permission prior to any caring procedure was obtained from only 23% of the cases (Table 2).

Data from the qualitative informants also indicated that there existed women’s concerns of respect dignity, and equity during the birthing process. While some informants discussed their experience of this dimension by considering the attendants’ moody behavior during service provision, others described it considering privacy experienced and the bias the attendants exercised during service provision. An informant shared that:

*… it was at noon. The environment sounds quiet. We approached the delivery ward, there was attendant taking nap. Mother-in-law asked, ‘doctor could you help us?’ He did not reply, he … said ‘you guys, you disturbed me.’ … , he asked me many questions, touch my belly, measure blood pressure, and other examination and said, ‘labor is too early and go back to home.’ I replied, I could not walk, it is long distance to go back to home, I have also pain. … . he shouted at me, ‘you guy, you can walk, are you a doctor to treat yourself?’*(Mother, 20’s – 30’s).

The narration revealed that the attendant did not welcome the mother. He was late to give the services and did not give appropriate answers to the mother’s questions. Rather, the attendant informed the mother to go to home without clear information of her status and what to do next if she leaves the facility. He also did not consider the mother’s concern.

The worry of privacy was reiterated by many of the informants. Almost all informants had concerns about privacy during the birthing process in the facilities. While some informants experienced low privacy while they were in the bed/examination rooms, others reported that their privacy was not respected both at bed/examination room as well as in the delivery room. Some informants shared that the prenatal ward rooms are shared rooms among laboring women waiting for delivery. There are also women’s companions in these rooms. Women discussed that their privacies were evaded when attendants took some examination on bed-sides (e.g., abdominal examination) and while the attendants discussed medical and pregnancy history with women. Informants shared that other laboring women and their fellow members would see their uncovered belly and listen their medical and health related histories told to the attendants.

Other informants discussed that the delivery wards have poor curtains or barriers so that people who got into the rooms could easily saw them while they were naked on the delivery coach. Regarding this, an informant shared that, “… .the room does not have door or any barrier. They laid me on the delivery couch and other staffs who come in and out of the delivery room watches my genital which is quite embarrassing.”

Some informants also shared that the presence of many professionals in a delivery ward was embarrassing as well as frustrating. While the presence of professionals together may help the attendants to share experiences and better understand the mothers’ context, mothers may not understand it the same way. A woman with previous home delivery experiences considered this experience as followed.

*In the prenatal room, there were many doctors, I guess they are students. One of them removed clothes from my belly and touches it around. He was telling them which I did not listen and understand. It may be ‘ferenjigna’- meaning English. Others also were saying something which I did not understand. I worried whether there is a problem, no one told me. While I was suffering, they were learning. They used me as ‘memaria’- meaning teaching aid* (mother, 30’s – 40’s).

The narration indicated that the mother understood that there was a discussion among students with their supervisor. The woman’s unclosed belly was demonstrated for teaching purpose. The discussion language was unfamiliar to the mother. She could not understand the points of the discussion. This worried her to know her status.

Informants also perceived that the timeliness and provision of delivery services at health facilities differ by companions of mothers and the presence/absence of social network professionals at health facilities. Informants described their experiences as well as others’ experiences they knew as follows.

*… they treat you depending on your background, i.e. it depends on the kind of family you come with and your appearance. If you come wearing nice clothes and accompanied by urban companions, they will give you priority. For mothers in torn clothes, then things are different. If you knew someone working within the facility, you will be favored by attendants* (Mothers, 20’s – 40’s).

The narration indicated that women perceived attendants lacked fairness in their service provisions. Women from well to do families and with networks within the health facilities are privileged to receive timely services. The data also revealed that poor mothers and mothers with limited social capital would not get timely services by attendants during facility delivery.

#### Emotional support

Emotional support refers to the psychological support, attentiveness to the feelings and needs of women in labor, and encouragement given by the attendant during the birthing process. Seventy three percent of mothers perceived that the attendants showed compassion to their concerns. About 47% of mothers experienced supportive staff and 35% reported that the attendants updated them the status and progress of their labor periodically. Only 2% of mothers reported that their preferred companions were with during the birthing process (Table 2).

Informants in the qualitative discussion have mixed perceptions of the quality of emotional support they experienced during the birthing process. While 16 out of 25 informants appreciated the human services of the attendants, others illustrated the indifferent and unresponsive services of the attendants. A mother who delivered her second child discussed that the attendants were considerate of her concerns and reassured her during the birthing process. She stated that:

*… we went there (*health facility*) at night. Labor was slow, no delivery till mid day. My husband went home to bring food. When he went out, there was fast cramp. I was alone and felt frightened. One of the attendants understood my context. She came close to me and said, ‘we are here to support you, do not wary. You will get your baby soon.’ … I had my son safely before my husband come back from home. I considered her as my sister* (mother 20’s – 30’s).

The narratives revealed that the attendants were supportive and close to mothers to the level to substitute family members. On the other hand, unsatisfying experience during the birthing process was reported. A mother shared her experience as:

*… I planned to deliver in health facility. When pain started, we went to the facility. After examination, the attendant informed me to stay in the waiting home (back yard of the facility). I stayed there three days. No one closely followed me the whole days, but they sometimes said how you are … from a distance. During late afternoon in the third day, the cramp became strong, my husband called the nurse. She came and … she said, ‘please do not disturb other women’ (*there are other two mothers in the waiting home). *During mid night, the pain became severe; we hesitated to call her. Fortunately, I got my daughter. My husband assisted me. The attendant came latter and shouted at me, ‘why you did not call me?’ … laughing* (mother, 30’s – 40’s).

The informants’ experience indicated that though the mother stayed around the facility to get prompt support from the attendants, there was limited contact with the attendant and the attendant was reluctant to provide services when needed. The attendant did not inform the mother on the status and progress of her labor. Such behaviors of the attendant made the couples to hesitate to call the attendant when they needed her support. The mother delivered without the assistance of the professional. Thus, the informant perceived that the attendants in the health facilities are indifferent of mothers concerns and the quality of delivery services at health facilities as poor.

Informants were discontented by the absence of their family members with them during labor and childbirth. They argued that during home delivery, relatives/friends are often with the laboring mother to support, encourage her, and pray for safe event. Such tradition is not allowed in health facilities. All informants shared that relatives/friends who accompanied them to health facilities were denied to be with them. The new settings with the unknown attendants increased their fear and anxiety while they were in labor. A mother who had her first birth stated that:

*… they* [the staff] *did not allow, even my mom to be with me. The pain was severe, I felt … between life and death. I wanted someone to hug me on the back, I shouted. The nurse did nothing, rather she said, please be quiet* (mother, 20’s – 30’s).

#### Level of Intrapartum care services

**Fig. 1:**
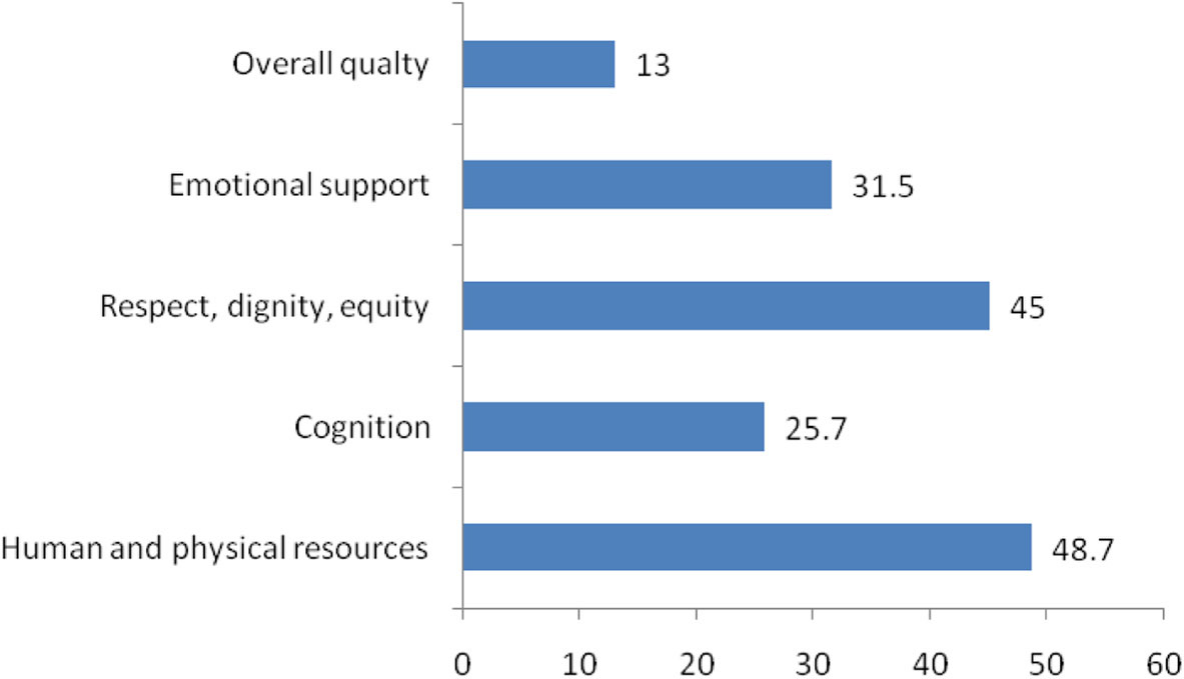
Percentage of mothers experienced good quality of intrapartum care services by dimensions of care (*n* = 378)

To determine the levels of quality of intrapartum care services experienced by mothers, we aggregated the responses of the quality issues assessed. Taking all dimensions together, only 13% of mothers experienced good quality of intrapartum care. Nearly half of mothers (48.7%) experienced good quality of human and physical resources and 45% experienced good quality of respect, dignity, and equity during the intrapartum period. Thirty one percent and one fourth of mothers experienced good quality of emotional support and cognition, respectively (Fig. 1).

**Fig. 2:**
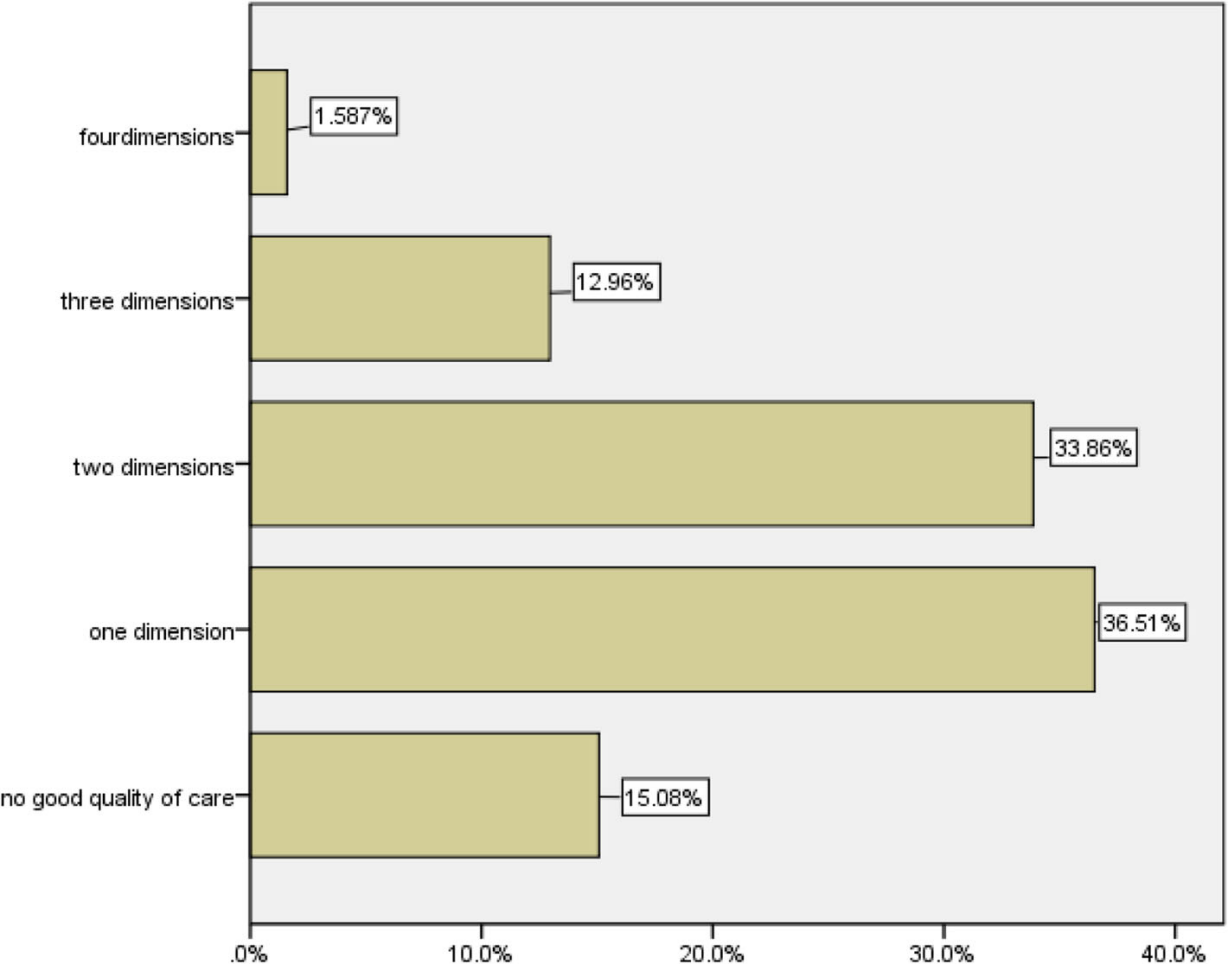
Percentage of mothers experienced good quality by the number of dimensions of care

Of all dimensions of intrapartum care assessed, about two in hundred mothers (1.6%), more than one in 10 mothers (13%), and about a third of mothers (34%) experienced good quality of services on four dimensions, three dimensions, and two dimensions of care, respectively. Thirty six percent of mothers experienced good quality of care on one dimension of the services. Fifteen percent of mothers did not experience good quality of services on any of the dimensions (Fig. 2).

Table 3 presented mothers’ overall experiences of the quality of intrapartum care services by their background characteristics. As presented in the table, mothers’ experience of quality of intrapartum care is comparable on many of their background characteristics. Greater proportion of women from rural areas than their urban counter parts (16.4% vs. 7.1%, χ2 = 6.67, df = 1, *p* < 0.05) and greater proportion of women who did not have antenatal care for their recent born children than those who had the services (20.3% vs.11.3%, χ^2^ = 4.01, df = 1, *p* < 0.05) experienced good quality of intrapartum care service during their recent delivery.

**Table 3:** Percentage distributions of respondents by background characteristics and perceived quality of intrapartum care experienced, *n* = 378

Background characteristics	Quality of care experienced		χ^2^
	Poor n(%)	Good n(%)	
**Age**			
18–25	139(84.8)	25(15.2)	1.33
≥ 26 years	190(88.8)	24(11.2)	
**Residence**			
Rural	199(83.6)	39(16.4)	6.67*
Urban	130(92.9)	10(7.1)	
**Marital status**			
Divorced	14(93.3)	1(6.7)	.55
Married	315(86.8)	48(13.2)	
**Educational status**			
No education	196(87.1)	29(12.9)	1.63
Primary education	73(83.9)	14(16.1)	
High school and above	60(90.9)	6(9.1)	
**Parity**			
One	97(86.6)	15(13.4)	4.18
Two to four	175(85)	31(15)	
Five and above	57(95)	3(5)	
**ANC use for the recent birth**			
Yes	274(88.7)	35(11.3)	4.01*
No	55(79.7)	14&20.3)	
**Intension of place of delivery**			
Home	36(85.7)	6(14.3)	.07
Health facility	293(87.2)	43(12.8)	
Total	329(87)	49(13)	

*p < .05

## Discussion

This study assessed the quality of intrapartum care experienced among women who had recent uncomplicated births in health facilities. The findings revealed that the quality of intrapartum care services from the perspectives of clients was low. Of all mothers participated in the study, only 13% (95% CI: 9.8–16.4) perceived that they experienced good quality of intrapartum care services. This finding is lower than earlier studies reporting the overall quality of delivery care at public health facilities was 54% [8], the proportion of women satisfied with delivery care was 62% in Amhara Region [24], and clients’ satisfaction with obstetric care in public health facilities in Jimma Zone, South West Ethiopia was 79.4% [28]. Variations in the findings could be due to different tools used for assessment, differences in the participants of the study, or the time frame when the research was conducted. For example, the participants in Tayelig and colleagues’ [24] study were women referred to hospitals due to obstetric problems. These women may overrate the quality of services they experienced comparing the complications they had before delivery and the relief they had after delivery. Moreover, women were interviewed while they were at health facility, hence they may not feel comfortable to report negative experience while they were in the facility [22]. The participants in the current study were women who had uncomplicated births at health facilities that exhaustion from complications may not have significant influence to judge the quality of services they experienced. Data were also collected at the community level where women may comfortably respond negative experiences without fear of the attendants.

This study assessed the quality of intrapartum care women experienced in health facilities considering four dimensions: their contact with and experience of human and physical resources; their cognition; the respect, dignity, and equity of care they receive in their stay in the facilities; and the emotional support they received. The findings revealed that 15% of mothers had poor experience on both dimensions of intrapartum care. Only two in 100 and two in 10 mothers respectively experienced good quality of services on four dimensions and three dimensions of intrapartum care. The findings are suggestive that the quality of intrapartum care in the district is very low.

Regarding contact with human and physical resources, only half of mothers (49%) participated in the study perceived that the quality of this dimension of care was good. Put it in other way, half of mothers experienced poor quality on this dimension of care. Poor quality issues identified in order of magnitude included absence clean toilet (57%), lack of available water (50%), unclean delivery rooms (47%), unclean delivery beds (42%), crowded labor room (30%), lack of electric light (19%), unclean labor ward (18%), and absence of attendants in the facilities when needed (10%). Majority of qualitative informants also described that they experienced bad odor in the labor wards. These findings implied that the government’s commitment of providing conducive environments for labor and delivery at health facilities [25] is not materialized. Though the magnitude differs, such poor quality of human and infrastructure during facility delivery was reported in poor town in India [23].

Cognition refers to the presence of client-provider information exchange during service provision [29] and the effectiveness of this information exchange depends on the existence of positive client-provider interaction [23]. In this study, provider-client communication and information exchange was found minimal. Only a quarter of mothers reported that they experienced good quality on this dimension of care during the birthing process. More specifically, about a quarter of the respondents were informed of what was happening to them and mothers’ questions were answered only for 27% of the cases during labor and delivery. In addition, assessments of health and obstetric conditions were informed only for 43% of cases. Informants also described that attendants showed bossy behavior and did not want to talk with mothers. Mothers were not informed of their health and obstetric status following examinations. There were contexts mothers often hesitated to ask questions expecting that the attendants may not give answers. These evidences suggest that the majority of women did not involve in decisions about their care and indeed are not even informed about what is happening to them and why. Such poor quality of communications between health care providers and their clients contribute to under-utilization of available health services [23, 30].

With respect to issues related to respect, dignity and equity, the quality issues assessed included privacy, clients’ participation in deciding caring procedures (consent), and staff politeness during communication. Less than half of mothers reported positive experience of this dimension of care. Regarding privacy, only half of survey respondents reported that their privacies were respected during service provisions. The problem of maintaining privacy was a big concern for most qualitative informants. Informants pointed out that their privacies were evaded while they were in the prenatal wards as well as in the delivery rooms because of the absence of separate prenatal/postnatal rooms for individual women, the adjacent classes serving different purposes, and absence and/or poor curtains or barriers in the delivery rooms. Similar findings were reported by earlier researchers that providers did not use curtains or visual barriers to protect privacy during child birth for 21.4% of mothers in Ethiopia [31] and providers did not use curtains or visual barriers to protect privacy during child birth for 58% of mothers and 84% of mothers required to share a bed in the postnatal ward in Tanzania [22]. Mothers in India were also examined in crowded places, without curtains to shield them [23]. Women with such experience may not be satisfied by the services [24] and did not use facilities for deliveries for subsequent deliveries [30].

In this study, less than a quarter of mothers (23%) were consented before any procedure performed in the course of labor. This is contrary to recommendations that “if interventions become necessary for valid indications, service providers must make the mother aware of the necessity as well as the risks of the intervention so that she can give informed consent” [32]. Hence, to make maternal health care services human, attendants should respect and consider women’s right to choose and participate in the decision making process [33].

Emotional support from relatives and friends as well as from the attendants during labor has benefit for a woman and her new born health [23, 34]. However, this study demonstrated that less than a third of the surveyed mothers (31%) reported good experiences of this dimensions of care for their recent delivery. The quality issues assessed included compassion for mother’s concerns, supportive staff, and presence of companions of mothers’ choice during labor and delivery, and updating the status and progress of labor. Greater proportion of women (70%) experienced low quality of this dimension of care.

Informants in the qualitative interview argued that relatives/neighbors as birth companions are not allowed to be with woman in a labor environment. Denying women this support is against evidence-based practice [35] and national guideline [25] and associated with dissatisfaction of women with facility childbirth services. Evidences indicated that institutional rules and strategies that restrict the presence of birth companions during labor and delivery in health facilities are barrier to humanized birth care [36] and this serve as a barrier for service utilization [37]. Thus, efforts underway to increase maternal health service utilization should consider the limitations of such restrictive institutional rules and strategies that deny presence of birth companions in the labor environment in health facilities.

In the current study, some qualitative informants discussed the lack of fairness in service provisions at health centers. Informants specified that the timeliness of service provision differs by companions of mothers (urban residents), women’s appearance (clothing), and the presence/absence of social network professionals at health facilities. In the quantitative finding, greater proportion of women from rural areas than their urban counter parts and greater proportion of women who did not have antenatal care for their recent born children than those who had the services experienced good quality of care service. These are significant findings and future research is needed to identify the extent to which these findings reflect a real differential experience of care or a different expectation of care. Whatever the relative balance, these are evidences that highlight an important quality concern.

This study had limitations. First, the findings are on the quality of intrapartum care services on offer from the clients’ perspectives. It did not tell us the quality of services provided by the facilities. Second, data were collected from women who had uncomplicated deliveries in health centers thereby limiting generalization of the findings to women who had complicated births and women who delivered in hospital settings. Third, the structured questionnaire used to quantify quality of intrapartum care experienced may not exhaustively address all indicators in those dimensions. To offset this limitation, we used pretested instrument and mixed methods design to obtain detailed information and complement each method.

## Conclusion

Our findings suggest that the quality of intrapartum care in health facilities was minimal. Only 13% of mothers had positive experience of the event. The setting for this study is typical of many locations in the country where incentivized access to service is increasing. The findings highlight the limitations of efforts which aim to increase facility births by increasing number of facilities and deploying professionals without a corresponding concern for quality. Mothers who experienced poor quality of services may be reluctant to use the services for subsequent deliveries as well as they may serve as barrier for service utilization by sharing their negative experiences for potential users. Thus, if Ethiopia is to succeed in its goal of increasing uptake of facility births and improved maternal health outcomes, strengthening a responsive health care system for mothers that will provide humane, respectful, and equitable care is a priority.

## Supplementary information

**Additional file 1.**

## Data Availability

The datasets analyzed during the study are available from the author on reasonable request.

## Abbreviation

ANC: Antenatal care
