# Supplementary material for: Quality of intrapartum care at health centers in Jabi Tehinan district, North West Ethiopia: clients’ perspective
Source: BMC Health Serv Res. 2020 May 19;20:439. doi: 10.1186/s12913-020-05321-3 (PMC7236140; doi:10.1186/s12913-020-05321-3)
Supplement: Supplementary file 1 — Additional file 1. [file 12913_2020_5321_MOESM1_ESM.docx]

## Additional file

## Structured Questionnaire

The purpose of this questionnaire is to collect data on the quality of intrapartum care services experienced by mothers during their recent births at health facilities. The data will help to know the levels of quality of delivery services on offer for mothers and their perspectives on the quality of services they experienced at health facilities. The findings will provide relevant information for service providers, decision makers, and other stakeholder on how well the institutions are functioning in providing client-centered delivery services and which aspects of care need to be improved. The information is needed for research purpose. The information given will be kept confidential.

Thank you in advance for your participation.

Respondent ID _________Health facility code ________ Kebele code _________

**Part I. Women’s Individual and childbearing-related attributes**

**Women’s individual attributes**

| Now I would like to ask you some question about you and your household. | | |
| --- | --- | --- |
| S. No. | Variable descriptions | Answers |
| 101 | How old are you? | Age in completed years ____ |
| 102 | Could you tell me your educational status? | 1. no education 2. primary education 1^st^ cycle (1- 4) 3. primary education 2^nd^ cycle (5 – 8) 4. high school (9 – 12) 5. tertiary education |
| 103 | Could you tell me your marital status at the time of delivery? | 1. never married, 2. Divorced 3. Widowed 4. married |
| 104 | Where is your usual residence? | 1. rural 2. urban |
| 105 | Where is your permanent living in relation to relatives? | 1. close to natal home, 2. close to nuptial home, 3. nuclear home away from both. |
| 106 | Where were you living at the time of your delivery? | 1. At natal home 2. At nuptial home 3. At own home |
| 107 | Where do most of your kin live? | 1. Neighbor 2. not neighbor |

**Women’s childbearing-related attributes**

| Now I would like to ask you some questions about your parity and maternal health service utilization | | | |
| --- | --- | --- | --- |
| S. No. | Variable description | | Optional answers |
| 108 | I understand that you had a delivery recently. How old is your last born child? If the response is “2” stop here. | | 1. ≤ 6 months  2. > 6 months |
| 109 | Could you tell me the birth order of your recent born child? | | 1.1^st^ birth skip to 113   1. 2^nd^ birth 2. 3^rd^ birth 3. 4^th^ birth 4. 5^th^ and above. |
| 110 | How many children do you have now who are living? | | Number of living cchildren _____ |
| 111 | Have you ever-used ANC services for the previous born children? | | 1. No 2. Yes |
| 112 | Have you ever-used SBAs services for your previous born children? | | 1. No 2. Yes |
| 113 | Have you had ANC visit(s) for your recent pregnancy? | | 1. No skip to 116 2. Yes |
| 114 | If the response to “113” is yes, where did you receive care? | | 1. health post 2. health center 3. private clinic |
| 115 | If the response to “113” is yes, number of ANC checkups, | | Number of ANC checkups__ |
| **Now I would like to ask your opinion on the following questions.** | | | |
| 116 | If you become pregnant again, where do you want to deliver? | 1. Home 2. Facility only if problems happened 3. Facility 4. Do not want to be pregnant again | |
| 117 | Where will you advise other Women to deliver? | 1. Home 2. Facility only if problems happened 3. Facility | |
| 118 | Where did you intend to give birth for your recent born child? | 1. At home 2. At health facility | |

**Part II. Experience of quality in delivery care**

| Now I would like to ask you about your experience of the services you had during labor and delivery in the health facility | | | |
| --- | --- | --- | --- |
| S. No. | Variables description  During labor and delivery of your recent born child in the health facility, which of the following happened to you? | Answer options | |
|  |  | 1= yes | 2 = no |
| 201 | I found clean and good-looking labor ward |  |  |
| 202 | Electric light is available in the facility |  |  |
| 203 | Labor room is well ventilated and not crowded |  |  |
| 204 | The delivery beds/coach is clean |  |  |
| 205 | The delivery room is clean |  |  |
| 206 | Water is available in the facility |  |  |
| 207 | The toilet in the facility is clean |  |  |
| 208 | Attendant (nurse/midwife) is available in the facility |  |  |
| 209 | Examination was taken at arrival |  |  |
| 210 | The attendant told the diagnoses (findings of the examination) |  |  |
| 211 | The attendant explains the activities done for mothers |  |  |
| 212 | The attendant answers mothers’ questions |  |  |
| 213 | The attendant advised health promotion (breast feeding, diet, immunization, etc.) |  |  |
| 214 | The attendant communicates politely while giving services |  |  |
| 215 | The prenatal ward and delivery rooms have screens or curtain to maintain privacy |  |  |
| 216 | The attendant informs and took permission prior to any procedure |  |  |
| 217 | The attendant was considerate of mother’s concerns |  |  |
| 218 | The staff in the ward are supportive |  |  |
| 219 | The attendant allowed companion of mother’s choice to be with her during labor and delivery |  |  |
| 220 | The attendant updates the status and progress of mother’s labor |  |  |

*Data collector’s Name _________________ signature ___________*

*Date of interview____________*

## Interview guide for qualitative data

Interviewee code ___________________

## Preliminaries

Now I would like to ask you some question about you and your household.

1. Age _____
2. Literacy status _____________________________________
3. Educational level (if literate) _______ ____________________
4. Marital status _________________________________
5. Residence /kebele ______
6. Birth order _______

**Childbearing-related attributes**

Now I would like to ask you some questions about your parity and maternal health service utilization

1. I understand that you had a delivery recently. How old is your last born child? Could you tell me the birth order of your recent born child? Parity -------.
2. Have you had ANC visit(s) for your recent pregnancy? How often you visited health facilities for the service? Where did you have follow ups?

**Experience of quality in delivery care**

I understand that you had a delivery at health facility. I would like to ask you about your experience of the services you had during labor and delivery.

1. When did you go to health facility for delivery? (During day time, night, etc). Whom you found around the delivery ward?
2. Tell me what the attendant(s) did for you? Probing items:

- Examination
- Specimen for laboratory test
- Whether the diagnoses explained
- Advice/suggestions on what to do and what not do

1. Could you tell me how the attendant was communicating with you during service provision? Manner of communication probing items:
   - - Asking questions
     - Listening responses
     - Feedbacks for questions
     - Politeness during communication
2. Could you tell me the characteristics of the laboring environment? Probing items:

- Characteristics of prenatal ward (number of beds, cleanliness, ventilation, adequacy of the room, screen/curtails)
- Characteristics of delivery room (delivery coach, ventilation in the room, cleanliness, wider room to walk around, screen/curtails)
- Characteristics of the postnatal ward ( beds, number of beds, cleanliness, silence)
- Availability of water and electric light
- Availability of toilet and its cleanliness

1. Could you tell me how the attendants deliver their services? Probing items:

- Whether caring procedures explained
- Consent before procedures
- Reassuring mothers labor progress
- Encouraging mothers labor efforts
- Updating progress of labor
- Mothers’ companion allowed

1. What services were given in the postnatal ward? Probing items:

- Mother’s and infant’s health check ups
- Breast feeding, hygiene,
- immunization

1. How did you explain the characteristics of the staff in the maternity ward?
2. If you become pregnant in the future, where will you give birth? Why?
3. Will you recommend the service for other pregnant women? Why?
4. Is there anything you would like to share me?

**Thank you very much for your time and important information you shared me.**

Date of interview ________________
